# Glucocorticoid Receptor β Overexpression Has Agonist-Independent Insulin-Mimetic Effects on HepG2 Glucose Metabolism

**DOI:** 10.3390/ijms23105582

**Published:** 2022-05-17

**Authors:** Claudia Sepúlveda-Quiñenao, Juan M. Rodriguez, Francisco Díaz-Castro, Andrea del Campo, Roberto Bravo-Sagua, Rodrigo Troncoso

**Affiliations:** 1 Laboratorio de Investigación en Nutrición y Actividad Física (LABINAF), Instituto de Nutrición y Tecnología de los Alimentos (INTA), Universidad de Chile, Santiago 7830490, Chile; claudia.sepulveda.q@gmail.com (C.S.-Q.); juan.rodriguez@inta.uchile.cl (J.M.R.); fdiaz@inta.uchile.cl (F.D.-C.); 2 Laboratorio de Fisiología y Bioenergética Celular, Escuela de Química y Farmacia, Facultad de Química y de Farmacia, Pontificia Universidad Católica de Chile, Santiago 7820436, Chile; andrea.delcampo@uc.cl; 3 Advanced Center for Chronic Diseases (ACCDiS), Universidad de Chile, Santiago 8380492, Chile; rbravosagua@inta.uchile.cl; 4 Laboratory of Obesity and Metabolism in Geriatrics and Adults (OMEGA), INTA, Universidad de Chile, Santiago 7830490, Chile; 5 Red de Investigación en Envejecimiento, Consejo de la Universidades del Estado de Chile (CUECH), Santiago 7830490, Chile

**Keywords:** glucocorticoid receptor β, liver, glucose, insulin, glycogen

## Abstract

Glucocorticoids (GC) are steroids hormones that drive circulating glucose availability through gluconeogenesis in the liver. However, alternative splicing of the GR mRNA produces two isoforms, termed GRα and GRβ. GRα is the classic receptor that binds to GCs and mediates the most described actions of GCs. GRβ does not bind GCs and acts as a dominant-negative inhibitor of GRα. Moreover, GRβ has intrinsic and GRα-independent transcriptional activity. To date, it remains unknown if GRβ modulates glucose handling in hepatocytes. Therefore, the study aims to characterize the impact of GRβ overexpression on glucose uptake and storage using an in vitro hepatocyte model. Here we show that GRβ overexpression inhibits the induction of gluconeogenic genes by dexamethasone. Moreover, GRβ activates the Akt pathway, increases glucose transports mRNA, increasing glucose uptake and glycogen storage as an insulin-mimetic. Our results suggest that GRβ has agonist-independent insulin-mimetic actions in HepG2 cells.

## 1. Introduction

Glucocorticoids (GCs) are steroid hormones that tune systemic energy metabolism to the circadian rhythm and psychological stress [1]. Psychological stress and awakening are highly energy-demanding states that stimulate GCs synthesis and release, increasing circulating glucose availability through hepatic glucose production (gluconeogenesis) and glycogen degradation [2]. Insulin, on the contrary, is a peptide hormone that drives glucose removal from the bloodstream via glucose uptake into cells and its storage as glycogen, especially in the liver and skeletal muscles [3]. These hypoglycaemic actions are mediated by the Akt kinase pathway, which ultimately induces the expression of glucose transporter 4 (GLUT4) and the glycogen biosynthetic enzymes Hexokinase II (mainly in skeletal muscle), Pyruvate Dehydrogenase Kinase 4 (PDK4), and Glycogen synthase. By contrast, excessive GC levels lead to hyperglycemia and hepatic insulin resistance, preluding diabetes mellitus and its complications [3].

GC signaling deploys a transcriptional program mediated by ubiquitous intracellular proteins, the Glucocorticoid receptors (GRs), which act as master transcription factors. Alternative splicing of the GR mRNA produces two isoforms, termed GRα and GRβ [4]. GRα is the canonical GC receptor, which mediates the GC actions through induction of Glucose 6-phosphatase (G6Pase) and Phosphoenolpyruvate carboxykinase (PEPCK), key enzymes of the gluconeogenic pathway [5,6]. In contrast, GCs do not bind to GRβ but act as a dominant-negative inhibitor of GRα-induced transactivation. Moreover, GRβ also has ligand-independent transcriptional activity [7].

Consistent with their diverging mechanisms of action, GRβ signaling closely recapitulates insulin. In cultured murine embryonic fibroblasts, GRβ mRNA reportedly increases after insulin stimulation, occurring in mice liver after fasting-refeeding. In both cases, GRα mRNA remained unchanged [8]. Accordingly, further studies showed that liver-specific GRβ overexpression reduces the mRNA levels of the gluconeogenic genes G6Pase and PEPCK, also PTEN, a protein phosphatase that opposes Akt signaling [9]. As a result, GRβ overexpression increases hepatic lipid accumulation, a marker of hepatic insulin resistance and the onset of fatty liver disease [10,11]. In addition, GRβ overexpression suppresses PTEN activity in the adipocyte cell line 3T3-L1, thereby augmenting Akt signaling and enhancing the insulin response [9].

This evidence strongly implies a regulatory role of GRβ on metabolic signaling. However, it remains unknown if GRβ regulates glucose uptake and storage and if it enhances the insulin effects in hepatocytes. Thus, this study aims to characterize the impact of GRβ on glucose uptake and storage, using the cell line HepG2 as an in vitro hepatocyte model.

## 2. Results

### 2.1. GRβ Overexpression Regulates GCs Target Genes and the Akt Signalling Pathway

**Figure 1:**
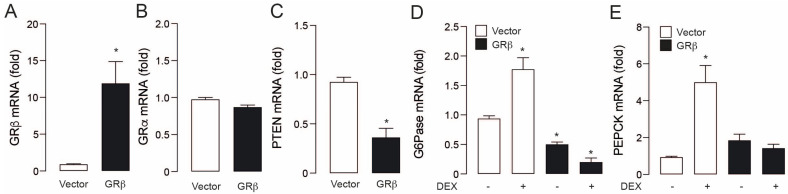
GRβ overexpression negatively regulates gene induction by GC in HepG2 cells. (**A**) GRβ mRNA levels. (**B**) PTEN mRNA levels. (**C**) G6Pase mRNA levels. (**D**) PEPCK mRNA levels. Data are presented as mean ± SEM. Student’s *t*-test was used in A−B−C. * *p* < 0.05 vs. Vector. Two−way ANOVAs followed by Sidak’s multiple comparisons test were used in (**D**,**E**). * *p* < 0.05 vs. Vector, *n* = 3.

To assess the role of GRβ in hepatocyte glucose homeostasis, we used the hepatocarcinoma-derived cell line HepG2, which has the presence of both GR isoforms [12]. We overexpressed the GRβ through transient transfection with a plasmid vector. After transfection, GRβ mRNA levels increased around 12 times compared to vector control (Figure 1A). This effect was specific because GRα mRNA did not show significant changes (Figure 1B). To study GRβ activity as a transcriptional repressor, we measured the mRNA levels of PTEN, G6Pase, and PEPCK. In PTEN and G6Pase, GRβ overexpression induced a baseline decrease in their mRNA (Figure 1C,D). To corroborate the negative regulation of GRβ over GRα targets, we also measured G6Pase and PEPCK mRNA in the presence or absence of the synthetic glucocorticoid dexamethasone (DEX). As expected, DEX treatment significantly augmented the mRNA levels of gluconeogenic genes, which was prevented by GRβ overexpression (Figure 1D,E). As previous reports [8,9], these data confirm that GRβ overexpression antagonizes GRα activity.

**Figure 2:**
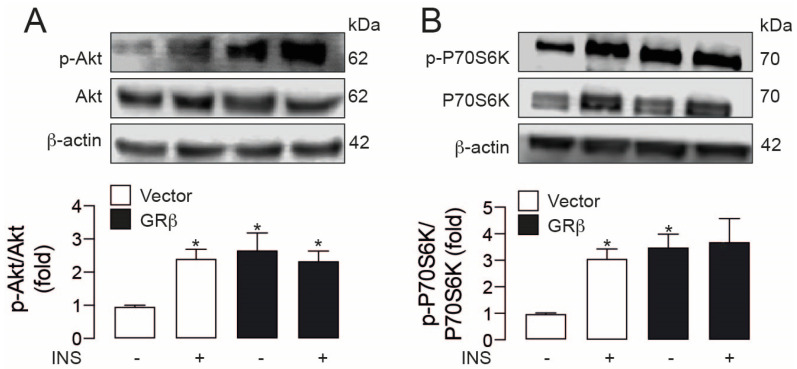
GRβ independently activates the Akt pathway in HepG2 cells. (**A**) Up: representative western blot images of phospho-Akt and Akt total, down: Western blot quantification of phospho-Akt and Akt total. (**B**) Up: representative western blot images of phospho-p70S6K and p70S6K total, down: Western blot quantification of phospho-p70S6K and p70S6K total. Data are presented as mean ± SEM. Two-way ANOVAs followed by Sidak’s multiple comparisons test were used. * *p* < 0.05 vs. Vector, *n* = 4.

Next, we assessed the effect of GRβ overexpression on the insulin-induced signaling cascaded in HepG2. As already described in other cell types [13], 30 min of insulin treatment leads to phosphorylation of both Akt (Ser^473^) and p70S6K (Thr^389^), an effect that was emulated by GRβ overexpression. Most notably, GRβ alone induced Akt and p70S6K phosphorylation in the same magnitude as insulin (Figure 2A,B). We did not detect a potentiation in the combination of both treatments. 

### 2.2. GRβ Overexpression Boosts the Insulin-Induced Increases in Key Glucose-Handling Genes

**Figure 3:**
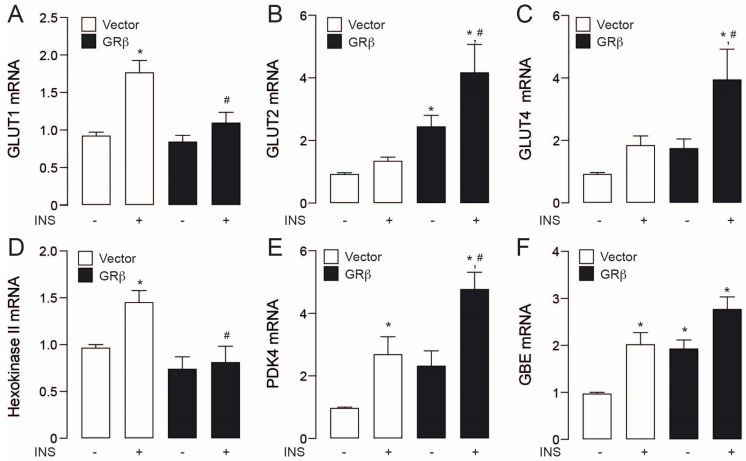
Effects of GRβ in glycolytic genes in HepG2 cells. (**A**) Glucose transport 1 (GLUT1) mRNA levels. (**B**) Glucose transport 2 (GLU2) mRNA levels. (**C**) Glucose transport 4 (GLUT4) mRNA levels. (**D**) Hexokinase II mRNA levels. (**E**) Pyruvate dehydrogenase kinase 4 (PDK4) mRNA levels. (**F**) GBE mRNA levels. Data are presented as mean ± SEM. Two-way ANOVAs followed by Sidak’s multiple comparisons test were used. * *p* < 0.05 vs. Vector, # *p* < 0.05 vs. Vector + INS, *n* = 3.

Apart from signalling events, we assessed the action of GRβ overexpression on genes related to glucose handling. We evaluated mRNA levels of GLUT1, a ubiquitous glucose transporter, GLUT4, which mediates insulin-induced glucose uptake and GLUT2, a liver- and β pancreatic-specific transport. Insulin only increased GLUT1 mRNA levels in empty vector cells, while the other two transporters were unchanged (Figure 3A–C). GRβ increased the mRNA levels of GLUT2 in comparison to cells empty vector stimulated with insulin (Figure 3B). Moreover, GRβ showed a boost effect in the GLUT2 and GLUT4 levels in insulin presence when compared with empty vector + insulin (Figure 3B,C). In the same way, to assess the interaction of GRβ and insulin, we assessed Hexokinase II mRNA levels, an enzyme that phosphorylates glucose, retaining it within the cell, PDK4, which prevents glucose oxidation by mitochondria, and the Glucose-branching enzyme (GBE) participates in glycogen synthesis. In these sets of genes, insulin treatment increased transcript levels on empty vector cells (Figure 3D,F). On the other hand, GRβ only increased GBE compared to empty vector cells (Figure 3F). GRβ overexpression and insulin treatment resulted in additive increments only in PDK4 mRNA compared with empty vector + insulin (Figure 3E).

### 2.3. GRβ Overexpression Activates the Insulin Signaling Pathway in HepG2 Cells

**Figure 4:**
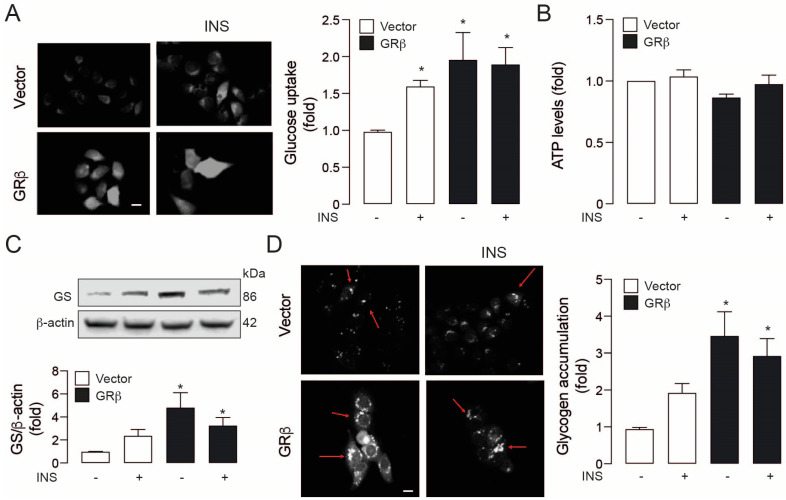
GRβ induces insulin-mimetic effects in HepG2 cells. (**A**) Left: 2-NBDG uptake representative images by live-cell epifluorescence microscopy. right: Glucose uptake quantification. (**B**) ATP levels. (**C**) Up: Glycogen synthase representative western blot images, down: Quantification of glycogen synthase western blot. (**D**) Left: Glycogen accumulation representative images by live-cell epifluorescence microscopy. right: Glycogen accumulation quantification. Data are presented as mean ± SEM. Two-way ANOVAs followed by Sidak’s multiple comparisons test were used. * *p* < 0.05 vs. Vector, *n* = 3.

Then, we addressed the functional changes of GRβ overexpression on glucose metabolism, starting with its uptake. GRβ overexpression increased glucose uptake similarly to insulin in empty vector cells (Figure 4A). However, there was no synergic effect, which is concordant with phospho-Akt and phospho-P70S6K protein levels (Figure 2). In all conditions (empty vector cells or GRβ overexpression), these changes did not increase intracellular ATP, suggesting that the increased glucose levels do not fuel energy metabolism (Figure 4B). These data agree with the previous result showing that the treatments that increase PDK4 levels switch glucose catabolism to fatty acid oxidation [14]. Finally, to explore this notion and determine the fate of the glucose intake, we measured the protein levels of the glycogen-generating enzyme, Glycogen synthase (GS), and glycogen accumulation in our model. At 3 h of insulin stimulation, we observed a slight no significant increase in GS in empty vector cells but significantly higher in the GRβ group compared to the control vector group (Figure 4C). These results led us to measure the glucose accumulation on our model (glycogen assay), where insulin stimulation on control vector cells do not induce an increase in glycogen storage. However, the GRβ overexpression caused a significant glycogen increment (Figure 4D), which is concordant with GS protein levels (Figure 4C). Conversely, we do not see a synergic effect with GRβ overexpression and insulin stimulation in GS protein levels and glycogen storage. 

## 3. Discussion

Here we show that GRβ overexpression can regulate the glucose metabolism acting like an insulin-mimetic decreasing gluconeogenic gene expression and increasing Akt phosphorylation, glucose uptake, and glycogen storage in the HepG2 cell line. Marino et al. found that the overexpression of hepatic GRβ in mice decreased liver gluconeogenic gene expression, associated with increased lipid accumulation and inflammatory markers [11]. Our results show that GRβ prevents the increase in G6P and PEPCK induced by DEX. Still, we did not observe a reduction if PEPCK in the GRβ transfected cells without DEX stimulation, which could be due to differences with the in vivo methodological approach that keep all the hormones (including glucocorticoids) working in the animal. In addition, He et al. showed in mice overexpressing the human GRβ a reduction in gluconeogenesis with no changes in hepatic morphology or hepatic lipid accumulation [10]. In this line, Stechschulte et al. showed that GRβ enhances insulin-induced proliferation by suppressing PTEN and activating Akt in adipocytes [9]. Akt is a central protein in insulin signaling, and we found that GRβ increases their phosphorylation and target protein p70S6K at the same magnitude as insulin. These data suggest that insulin action and pathway also should be regulated by GRβ. In this sense, early work showed that insulin increase the GRβ in the liver of mice [8], suggesting a bidirectional regulation between GRβ and insulin.

A critical step in glucose metabolism is their transport from extracellular space into the cytoplasm, regulated by the glucose transporter proteins (GLUT) [15]. We found that GRβ up-regulates the GLUT2 mRNA levels. This response increased in the presence of insulin, suggesting an increase in glucose uptake in the basal state and insulin-stimulated. GLUT1 mRNA was not regulated for GRβ, which contrasts with the data shown by Marino et al., who found a decrease in GLUT1 mRNA in the liver [11]. These differences could be explained because Marino et al. used a murine model, while our in vitro model is from human hepatocarcinoma, which on the one hand, are different species. On the other hand, HepG2 is a cancer cell line that could generate a different expression of glucose transporters, mainly GLUT1 [16]. GLUT4 mRNA was not regulated for GRβ. However, the mRNA levels of GLUT4 were higher in GRβ plus insulin condition, suggesting that insulin together with GRβ can co-regulate gene transcription through an unknown mechanism, which could be an exciting investigation topic. 

We found that GRβ increased glucose uptake to the same extent that insulin, and when we measured glycogen levels on the cells, we found an increase in the glycogen accumulation with GRβ associated with an increase in the glycogen synthase proteins levels, suggesting a regulation of anabolic processes, which is contrary to the GRα actions. Thus, a direct relationship between the glucocorticoid receptor and the mitochondria has been described [17,18]. However, whether the isoform β could negatively or positively regulate the mitochondrial function is unknown. On the other hand, our glycogen results contrast with Marino et al., showing a not significant reduction in liver glycogen of 21% [11]. However, the animal vs. cell model difference could account for the contradictory results. Thus, more studies are necessary to understand the insulin-mimic effects of GRβ in the liver but also in other tissues such as skeletal muscle and adipose. Several pieces of evidence demonstrated the role of GRβ in cellular processes like metabolism, inflammation, cell migration, cell growth, and apoptosis [19]. Thus, studying their cellular and physiological function is necessary to understand the intricate plethora of effects of glucocorticoid receptors.

In conclusion, GRβ overexpression activates the central canonical regulators of the insulin signaling cascade (Akt and P70S6K), increases glucose uptake, and glycogen storage in the HepG2 cell line, showing an additive effect with insulin in some markers of glucose handling, suggesting a converge in the response, ultimately GRβ acting as insulin-mimetic. 

## 4. Materials and Methods

### 4.1. Cell Culture and Treatments

HepG2 cells (ATCC HB-8065) were cultured in DMEM high glucose (Thermo Scientific, Waltham, MA, USA) supplemented with 10% (*v*/*v*) fetal bovine serum (FBS), 1% (*v*/*v*) penicillin-streptomycin (Biological industries, Kibbutz Beit-Haemek, Israel) solution in a humidified atmosphere with 5% CO_2_ at 37 °C. When cells reached 80% confluence, they were transfected with a plasmid-encoded the human GRβ kindly donated by John Cidlowski or an empty vector using lipofectamine 2000 (11668019, Thermo Scientific, Waltham, MA, USA) in optiMEM (31985-070, Thermo Scientific, Waltham, MA, USA) medium overnight. Then, the optiMEM medium was removed and replaced with fresh DMEN + 10% FBS medium for 1 h. After, cells were stimulated with dexamethasone (D2915, Sigma, St. Louis, MO, USA) 1 μM for 24 h or insulin (I9278, Sigma, St. Louis, MO, USA) 100 nM for 30 min or 3 h. 

### 4.2. Immunoblotting

Protein samples of HepG2 cells were prepared in NP40 lysis buffer supplemented with protease (04693159001, Roche Diagnostics, Basel, Switzerland) and phosphatase inhibitor (04906845001, Roche Diagnostics, Basel, Switzerland). Proteins were resolved in SDS-polyacrylamide gels and then subjected to immunoblotting using antibodies specific for phospho-p70S6K (9205, Cell Signaling, Danvers, MA, USA), p70S6K (2708, Cell Signaling, Danvers, MA, USA), phospho-Akt (9272, Cell Signaling, Danvers, MA, USA), Akt (9271, Cell Signaling, Danvers, MA, USA), glycogen synthase (GS-7H5, Santa Cruz Biotechnology, Dallas, TX, USA) and β-actin (Sigma, A2228, St. Louis, MO, USA). After, membranes were incubated with secondary antibodies anti-mouse (402335, Sigma, St. Louis, MO, USA) or anti-rabbit (401315, Sigma, St. Louis, MO, USA) conjugated to horseradish peroxidase and the ECL detection kit (Biological industries, Kibbutz Beit-Haemek, Israel).

### 4.3. Real-Time PCR (RT-PCR)

Total RNA was obtained from HepG2 cells using TRIzol reagent (15596-026, Thermo Scientific, Waltham, MA, USA). According to manufacturer protocol, cDNA was prepared from 1 μg of RNA using SuperScript II enzyme (A13268, Thermo Scientific, Waltham, MA, USA). Real-time PCR was performed using Eco Real-Time PCR System (Illumina, San Diego, CA, USA), using Fast SYBR Green Master Mix (4385370, Thermo Scientific, Waltham, MA, USA). The primers sequences are presented in Supplementary Table S1. Results were expressed to the geometric mean expression of three of the most stable housekeeping genes (Gapdh, Ywhaz, and β-actin). The ΔΔCT method was used for relative quantification analysis.

### 4.4. 2-NBDG (2-(N-(7-Nitrobenz-2-oxa-1,3-diazol-4-yl)amino)-2-deoxyglucose) Uptake and Accumulation

For glucose uptake, HepG2 cells were serum-starved for 3 h; then cells were stimulated with insulin 100 nM for 30 min and incubated with 2-NBDG 300 μmol/L for 15 min at 37 °C as previously described [13]. Imaging took place in an inverted microscope (Nikon Ti, Tokyo, Japan) equipped with a 40× NA 1.3 oil objective. Emission over 515 nm was collected using long-pass filters and digitalized by a cooled charge-coupled device camera (Hamamatsu ORCA 03, Hamamatsu, Japan). Images were quantified by ImageJ software (NIH, Bethesda, MD, USA). Also, we determined glycogen formation through the 2-NBDG accumulation, HepG2 cells were stimulated for 3 h with insulin, and images were taken as described previously.

### 4.5. Statistical Analysis

Data are presented as mean ± SEM of at least 3 independent experiments. The student’s *t*-test was performed to compare two groups. Multiple groups were analyzed using two-way ANOVAs followed by Sidak’s multiple comparisons test. All analyses were performed with GraphPad software (San Diego, CA, USA). *p*-value < 0.05 was considered statistically significant.

## Data Availability

Not applicable.

## Supplementary Materials

The following supporting information can be downloaded at: https://www.mdpi.com/article/10.3390/ijms23105582/s1.
