# Supplementary material for: Glucocorticoid Receptor β Overexpression Has Agonist-Independent Insulin-Mimetic Effects on HepG2 Glucose Metabolism
Source: Int J Mol Sci. 2022 May 17;23(10):5582. doi: 10.3390/ijms23105582 (PMC9141770; doi:10.3390/ijms23105582)
Supplement: Supplementary file 1 [file ijms-23-05582-s001.zip › ijms-1668839-supplementary.pdf]

Supplementary Table S1. List of Primers

Gene	Forward primer	Reverse Primer
GRβ	5’ ACTCTTGGATTCTATGCATGAAAATG 3´	5’ TGTGTGAGATGTGCTTTCTGGTTT 3’
PTEN	5’ GGCTAAGTGAAGATGACAATC 3’	5’ A GTTACTCCCTTTTTGTCTCTG 3’
G6P	5’ CCGAGCTTSCTSCACTTTTC C 3’	5’ GGTCGATGACCAAATAATATCC 3’
PEPCK	5’ ATTCTGGGTATAACCAACCC 3’	5’ GTTGATGGCCCTTAAATGAC 3’
SLC2A1	5’ ACCTCAAATTTCATTGTGGG 3’	5’ GAAGATGAAGAACAGAACCAG 3’
SLC2A2	AGAAGATTAGACTTGGACTCTC	5’ GTGACCTTATCTTCTGTCATTG 3’
SLC2A4	5’ CCATTGTTATCGGCATTCTG 3’	5’ ATTCTGGATGATGTAGAGGTAG 3’
Hexokinase II	5’ GAAAGCAACTGTTTGAGAAG 3´	5’ CAATGTCTGAGATGTCTTTGG 3´
GBE	5’ ATTGTGCTAGATTCAGATGC 3’	5’ CACCAAAAGAGAATAGGGAC 3’
GAPDH	5’ CTGCCCCCTCTGCTGATG 3’	5’ TCCACGATACCAAAGTTGTCATG 3’
β-Actin	5’ GGACTTCGAGCAAGAGATGG 3’	5’ AGCACTGTGTTGGCGTACAG 3’
YWHAZ	5’ TGCTTCACAAGCAGAGCA 3’	5’ GTTAAGGGCCAGACCCAGTC 3’
PDK4	5’ CTTGGGAAAAGAAGACCTTAC 3’	5’ GTGCAGTGGAGTATGTATAAC 3’
